# Effect of High-Frequency Electric Pulse on the Solidification Microstructure and Properties of Hypoeutectic Al-Si Alloy

**DOI:** 10.3390/ma17020468

**Published:** 2024-01-18

**Authors:** Jianjun Guo, Fang Wang, Shijie Zhang, Yifan Zhou, Lin Zhu

**Affiliations:** 1 School of Material Science and Technology, Taiyuan University of Science and Technology, Taiyuan 030024, China; jjg@stu.tyust.edu.cn (J.G.); s202114210078@stu.tyust.edu.cn (S.Z.); zhulin@tyust.edu.cn (L.Z.); 2 Hebei Province Automotive Safety Parts Technology Innovation Center, Baoding 072750, China; 15203431569@163.com

**Keywords:** high-frequency electrical pulse, Al-9Si aluminum alloy, microstructure, mechanical property

## Abstract

The effects of different pulse frequencies on the microstructure grain size and solid solubility of Al-9Si alloy were systematically investigated using OM, SEM, and EDS. The impact on the mechanical properties of the alloy was analyzed using a micro-Vickers hardness tester and multifunctional friction tester. During solidification, the Al-9Si alloy is exposed to high-frequency electric current pulses with a current density of 300 A/cm^2^ and frequencies of 0 Hz, 500 Hz, 1000 Hz, and 2000 Hz. The experimental results show that the Lorentz force also increases as the high-frequency pulse frequency increases. Intense electromagnetic stirring leads to grain refinement. However, as the pulse frequency continues to grow, the combined effect of Joule heating and Lorentz force results in an enlargement of the melt zone and an increase in grain size. At a pulse frequency of 1000 Hz, the eutectic structure size of the Al-9Si alloy is optimal, with the average size being reduced to 13.87 μm and a dense distribution, effectively eliminating primary Si. The EDS results revealed that the high-frequency pulse led to a more uniform distribution of Si elements within the matrix, and the solid solubility of Si in the α-Al matrix increased to a maximum value of 1.99%, representing a 39.2% increase. At a pulse frequency of 1000 Hz, the sample demonstrates the most favorable mechanical properties, with the friction coefficient reaching a minimum value of 0.302, representing a 37.7% decrease in the average friction coefficient. The results demonstrate that high-frequency pulsing is an effective method for enhancing the mechanical properties of Al-9Si alloy.

## 1. Introduction

The hypoeutectic Al-Si alloy is a crucial structural material and has garnered significant attention due to its favorable mechanical and thermodynamic properties. It boasts a low thermal expansion coefficient, excellent wear resistance, and high specific strength, making it widely utilized in diverse industries such as automotive, aerospace, and electronics [1,2,3,4,5]. The most commonly used method for modifying aluminum alloys is through the use of chemical modifiers, such as Na, P, Sr, rare earth elements, and a combination of multiple elements, which can refine the grain structure, improve distribution, and homogenize the microstructure, thus significantly enhancing performance. Hong et al. [6] found that the intermediate alloy Al-5Ti-1B can effectively refine the dendritic α-Al grains, attributed to the excellent heterogeneous nucleation ability of TiB2 and Al3Ti particles. However, for aluminum–silicon alloys with a silicon content exceeding 4%, the refining effect of titanium-containing refiners decreases due to the toxic reaction between titanium and silicon. In the case of Al-Si alloys, the microstructure depends not only on its dendritic structure but also on the size and morphology of the eutectic Si particles [7,8,9]. Researchers use two or more refining agents and modifiers to refine α-Al and eutectic Si in Al-Si alloys [10].

Traditional modification methods can be complemented by high-frequency electric pulse as a novel technique for controlling solidification microstructures. This method enables the overall adjustment of the size and morphology of α-Al, primary Si, and eutectic Si. It is characterized by a high-energy, non-equilibrium input periodically and repetitively, allowing for rapid heating and cooling, attracting widespread attention from scholars [11,12,13,14,15]. In the solidification of hypoeutectic aluminum–silicon alloys, high-frequency electric pulse can be used to selectively control the solidification microstructure by regulating the morphology and kinetic properties of the phase transformation interface, thus optimizing the comprehensive mechanical properties of the alloy [16]. Wang et al. [17] and many other scholars have researched the influence of high-frequency electric pulse on the solidification microstructure of hypoeutectic aluminum–silicon alloys and found that it can enhance the strength and plasticity of the alloy by combining Mg2Si phases and Mg4(Si, Cu) clusters. Edry et al. [18] discovered through theoretical simulation and experimental research that high-frequency electric pulse significantly impacts the size and distribution of grain size and morphology during alloy solidification, leading to grain refinement and directional growth. Electric pulse treatment, as a new efficient and energy-saving method, has been widely used to improve the microstructure and enhance the comprehensive mechanical properties of various alloy materials.

To further investigate the impact of high-frequency pulses on the microstructure of hypoeutectic aluminum alloy, this study selected the commonly used Al-9Si hypoeutectic Al-Si alloy as the research subject. This study compared the samples’ microstructure characteristics and mechanical properties before and after high-frequency pulse treatment. It analyzed the influence of high-frequency electric pulses on the solidification microstructure of hypoeutectic Al-Si alloy and discussed the underlying mechanism. This research provides a theoretical foundation for the continued development of high-frequency electric pulse technology in preparing hypoeutectic Al-Si alloys.

## 2. Materials and Methods

The industrial pure Al ingot with a 99.8% purity (mass fraction, as indicated below) was combined with a compacted block of Si (99.99% purity) powder using a resistance furnace and hydraulic press to create an intermediate alloy. From there, the production of hypoeutectic Al-9Si alloy began, containing less than 12.6 wt.% Si. The composition and content of the material elements are detailed in Table 1. Throughout the process, temperatures were monitored and recorded with an infrared thermometer, and all samples were subject to consistent cooling conditions with a controlled total mass deviation of ±0.5 g.

**Figure 1:**
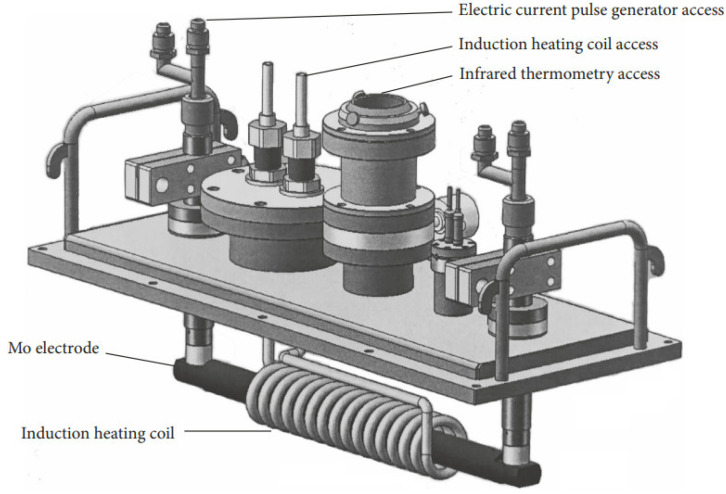
Diagram of experimental device.

**Figure 2:**
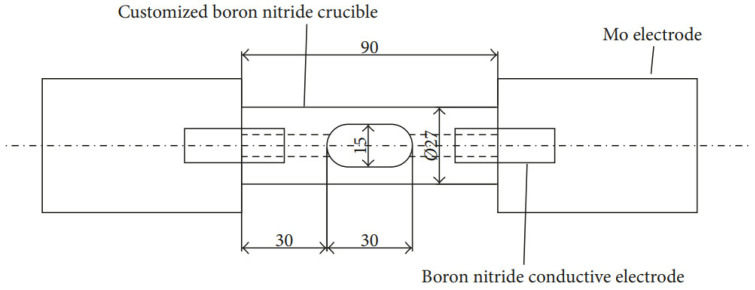
Size diagram of electrode crucible.

The process of preparing the hypoeutectic Al-9Si alloy under the influence of high-frequency pulses is illustrated in Figure 1. The equipment used consists of a pulse power supply, vacuum system, and an induction heating system, which has an output power of 35 kW and a working frequency of 20 kHz to 50 kHz. The induction-heated copper coil has a diameter of 6 mm and is supplemented with a temperature-measuring system and pressure device. During the experiment, the samples were divided into four groups and subjected to high-frequency pulses of 0 Hz, 500 Hz, 1000 Hz, and 2000 Hz, respectively. The samples were placed in customized cylindrical boron nitride crucibles with a diameter of 7.5 mm and a length of 30 mm, as shown in Figure 2. The furnace was then evacuated from 10^−4^ Pa to 10^−3^ Pa and filled with argon to −0.05 MPa. The crucible was connected horizontally on both sides to the molybdenum electrode of the pulse power supply through the boron nitride conducting electrode. The sample was melted using an induction heating device, maintained at 700 °C for 10 min, and then subjected to high-frequency electrical pulses for 30 s before stopping heating. The cooling curve of the temperature sample was observed, and once it entered the slow cooling stage, the high-frequency pulse power supply was turned off. Finally, the sample was aerated into the chamber to return to atmospheric pressure after cooling within the furnace.

The prepared hypoeutectic Al-9Si sample was sectioned along the cross-section, polished successively with 600, 1000, 1500, and 2000 mesh sandpaper, and then polished using a polishing machine. Subsequently, the clean surface was treated with Keller reagent (1 vol% HF + 1.5 vol% HCl + 2.5 vol% HNO_3_ + deionized aqueous solution) for corrosion, followed by rinsing with alcohol. Finally, the surface was blow-dried until smooth, and the microstructure was observed.

The metallographic structure of the sample was examined using a ROLYVER.MET metallographic microscope. Additionally, the samples were analyzed using an electron microscope and EDS on a JSM-6510 SEM and OXFORD X-MaxN spectrometer, respectively.

## 3. Results and Discussion

### 3.1. Structure Analysis

**Figure 3:**
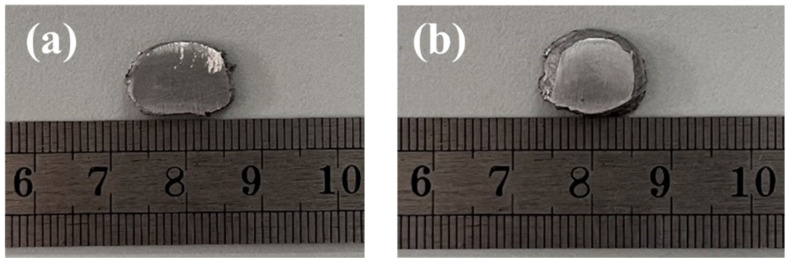
Macroscopic microstructure of hypoeutectic Al-9Si alloy samples: (**a**) untreated; (**b**) application of high-frequency pulse.

High-frequency pulses at frequencies of 0 Hz, 500 Hz, 1000 Hz, and 2000 Hz were applied to the solidification process of hypoeutectic Al-9Si alloy melt, and the macroscopic and microscopic solidification structures of the specimens were observed. Figure 3 shows the macroscopic morphology of hypoeutectic Al-Si solidification specimens before and after the application of high-frequency pulses. As shown in Figure 3a, obvious layering phenomena were observed on the surface of the solidification specimens under the condition of no high-frequency pulse application. However, as shown in Figure 3b, after high-frequency pulse treatment, the layering phenomenon disappeared, and the surface of the specimens appeared smooth with a metallic luster, indicating that high-frequency pulse current treatment can significantly improve the homogenization of hypoeutectic Al-9Si alloy specimens.

**Figure 4:**
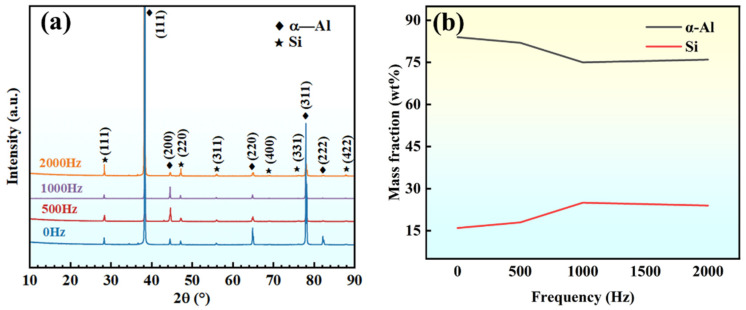
XRD results (**a**) phase; (**b**) phase content.

The phase composition and content of the hypoeutectic Al-9Si alloy after applying high-frequency pulses at different frequencies are shown in Figure 4. As seen in Figure 4a, the microstructure mainly comprises α-Al and Si phases. Quantitative analysis revealed that the relative content of the two phases changed with the pulse frequency variation, as shown in Figure 4b. When no frequency was applied, the Si phase content was only 16 wt.%. As the pulse frequency increased, the Si phase content also increased. When the frequency of 1000 Hz was applied, the Si content reached its highest value, increasing to 25 wt.%.

**Figure 5:**
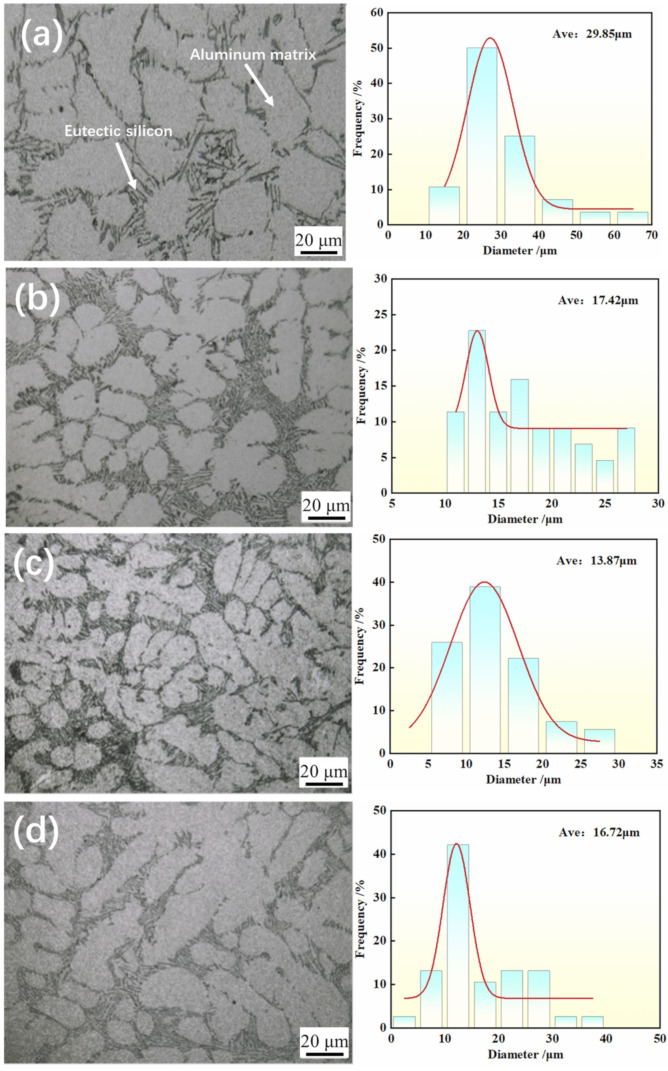
Microstructure of hypoeutectic Al-9Si alloy after application of different frequencies: (**a**) 0 Hz; (**b**) 500 Hz; (**c**) 1000 Hz; (**d**) 2000 Hz.

Figure 5 shows the microstructures of hypoeutectic Al-9Si alloy after applying high-energy pulses at different frequencies. As shown in Figure 5a, it can be observed that without using a high-frequency pulse current during the solidification process of the alloy, the α-Al matrix in the solidified structure exhibits sharp angles and a disordered distribution, which may lead to localized stress concentration. Additionally, at this stage, the grain size is relatively large. The combination of unfavorable grain morphology and size results in a decrease in the mechanical properties of the material. With the increase in frequency, as shown in Figure 5b, the α-Al exhibits a curved and transitional shape, and grain blunting becomes evident. Upon increasing the high-frequency pulse to 1000 Hz (Figure 5c), the average grain size decreases from 29.85 μm to 13.87 μm. In Figure 5d, as the pulse frequency continues to increase to 2000 Hz, the majority of the α-Al phase appears spherical and short rod-shaped, and the average grain size increases to 16.72 μm.

**Figure 6:**
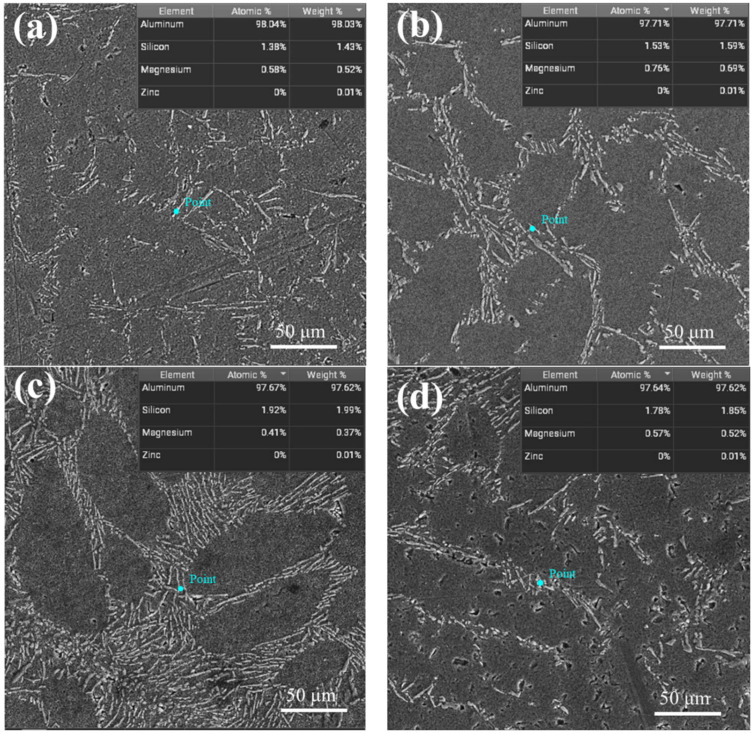
SEM images of solidification microstructure of hypoeutectic Al-9Si alloy after application of different frequencies: (**a**) 0 Hz; (**b**) 500 Hz; (**c**) 1000 Hz; (**d**) 2000 Hz.

Figure 6a–d show the SEM morphology and energy spectrum results of hypoeutectic Al-9Si alloy under the action of different frequency electric pulses at a constant current density. In Figure 6a, when no high-frequency pulse is applied, the eutectic structure is sporadically distributed and disordered, with a small amount of solute Si dissolved in the α-Al phase and an average size of 17 μm for primary Si. In Figure 6b, after applying a high-frequency pulse of 500 Hz, the eutectic Si exhibits a continuous, finely fragmented, or filamentous distribution on the matrix due to the promotion of faster and more uniform mixing of the melt by the high-frequency pulse. Additionally, the high-frequency pulse promotes nucleation and the growth of primary Si, observing the eutectic structure and a small amount of primary Si with an average size of 8 μm. The energy spectrum results show that the Si content is 1.59%, and the solid solubility of Si has increased by 11.2%. When the high-frequency pulses are increased to 1000 Hz, as shown in Figure 6c, eutectic Si increases. It is observed that the eutectic Si is refined into a point-like and fine fibrous morphology with a uniform, dense, and directional arrangement. At this point, the solid solubility of Si in the α-Al matrix reaches a maximum of 1.99%, representing an increase of 39.2% compared to the untreated sample. The primary Si is almost completely absent. In Figure 6d, when the high-frequency pulses continue to increase to 2000 Hz, it is evident that the amount of eutectic structure decreases and becomes intermittently distributed. The content of primary Si increases, while the solid solubility of Si in the α-Al matrix remains high, reaching 1.85%. Overall, it is observed that, at a frequency of 1000 Hz, the microstructure of hypoeutectic Al-9Si alloy exhibits the most effective refinement and modification of the eutectic Si. The pulsed current significantly enhances the solute trapping effect in the solidification structure of hypoeutectic Al-9Si alloy. As the pulse frequency gradually increases, the solute trapping effect becomes more pronounced.

### 3.2. Grain Refinement Mechanism

Some viewpoints suggest that the main reasons for grain refinement and equiaxed dimples caused by electric current and electromagnetic stirring are that the formation of crystal embryos promotes heterogeneous nucleation [19,20] and dendrite fragmentation [21]. Under high-frequency pulses, the Lorentz force increases, and the strong electromagnetic stirring action refines the grains, producing more spherical and elliptical α-Al phases, tending towards equiaxed. However, as the pulse frequency increases, the Joule heating effect strengthens. With the combined action of the Lorentz force, the dendrites at the solid–liquid interface fracture or remelt, increasing the length of the melt zone, and the average grain size increases. Furthermore, the solidification process of the alloy differs from that of pure metals. Based on the basic principles of atomic clusters, the alloy melt comprises clusters and disordered atoms [22]. Because the arrangement of atoms within the clusters is more regular than in the disordered liquid phase, it offers less hindrance to the movement of electrons [23,24]. Therefore, when an electric current flows through the metal melt, the additional Gibbs free energy difference caused by clusters and the disordered liquid phase due to the high-frequency pulsed current is:

(1) $$∆G^{a}=K_{1}j^{2}\xi V$$

where $K_{1}$ is a constant related to the material; j represents the current density; $\xi =\left(\sigma_{0}-\sigma_{n}\right)/\left(2\sigma_{0}+\sigma_{n}\right)$, where $\sigma_{0}$ and $\sigma_{n}$ are the conductivities of the disordered melt and the nuclei, respectively; and V is the volume of the nuclei. Typically, the conductivity of the clusters is higher than that of the disordered melt, with $∆G^{a}<0$. According to classical solidification theory, the average number $N_{n}$ of clusters with n atoms in a metal melt can be obtained by the following equation [25]:

(2) $$N_{n}=N_{l}ecp\left(-\frac{∆G_{n}}{k_{B}T}\right)$$

where $N_{l}$ represents the total number of atoms in the melt; $∆G_{n}$ is the difference in free energy between a single atom in a cluster and a single atom in the disordered liquid; $k_{B}$ is the Boltzmann constant; and T is the absolute temperature of the melt. When there is a high-frequency pulsed current in the melt, $∆G_{n}$ is composed of the free energy difference $∆G_{n}^{0}$ when no current is passing through and the additional Gibbs free energy difference $∆G_{n}^{a}$ between clusters and the disordered liquid phase caused by the current passing through, as in Equation (3):

(3) $$∆G_{n}=∆G_{n}^{0}+∆G_{n}^{a}$$

Considering the above equation, it can be inferred that the current increases the number of atomic clusters $N_{n}$ in the melt. As in Equation (4), the increase in the concentration of atomic clusters inevitably leads to an increase in the nucleation rate I in the subsequent nucleation process, resulting in the refinement of grain size. $\frac{dn}{dt}$ represents the adsorption rate of atoms.

(4) $$I=N_{n}\frac{dn}{dt}$$

Elements will form unstable bonds with surrounding elements in a metallic bonding manner. Under ideal conditions, they can be regarded as colloidal particles of the same size. Based on this, electric pulses decrease the outermost layer potential of the colloidal particles, reducing the bonding energy between the colloidal particles and the metal particles. As a result, the atoms in the colloidal particles are more likely to bond with the atoms in the metal particles, forming nuclei after bonding and thus increasing the effective nucleation in the melt, ultimately refining the grain size.

### 3.3. Solid Solution Mechanism

**Figure 7:**
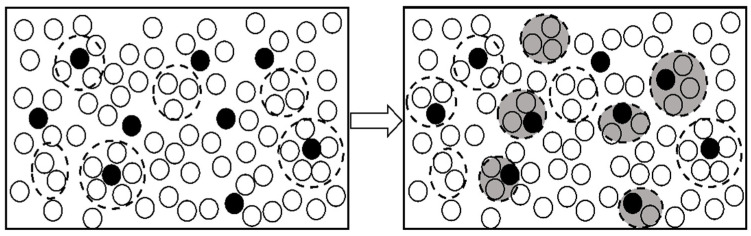
Liquid structure before and after high-frequency pulse action.

Many studies by scholars [26,27,28] have shown that in liquid alloys, due to the difference in electric polarity, one type of ion exhibits a positive ion effect. At the same time, the other reveals a negative ion effect. The solvent clusters (solvated solute atomic clusters) show electrical neutrality. According to the thermodynamic properties, solvent clusters are more stable than solute clusters and solvent clusters. At the atomic level, the atoms within the clusters cannot move freely, and the clusters move as a whole. The atoms within the clusters retain most of the information of the solid phase. When high-frequency pulses are introduced, smaller solvent clusters may potentially form new, smaller-volume solvated clusters with solute atoms, leading the system from one equilibrium state to another. Applying electric pulses to the melt disrupts the original equilibrium of atomic clusters and leads to a structural transformation. At a specific thermodynamic state, larger-scale clusters become less stable while smaller-scale clusters that satisfy specific magic number characteristics dominate, as shown in Figure 7.

The application of pulses reduces the number of larger-sized clusters in the melt, resulting in an increase in the cluster surface area. Assuming σ represents the interface energy between atomic clusters and the free atomic liquid, then the interface energy of an atomic cluster with a radius of R is given by:

(5) $$G_{\sigma }=4\pi R^{2}\sigma$$

After the application of a pulse, the cluster ruptures into n smaller atomic clusters, for ease of understanding, assuming their radii are equal and the total volume of the cluster remains unchanged. Letting r denote the radius, we have *r* = $r=n^{\frac{1}{3}}R$. The total surface free energy after the rupture can be calculated as follows:

(6) $$G_{\sigma }^{\prime }=4\pi n^{\frac{2}{3}}R^{2}\sigma$$

From the above two equations, it can be inferred that the interfacial energy increases by Δ*G* = $∆G=4\pi (n^{\frac{2}{3}}-1)R^{2}\sigma$. The diffusion of solute needs to overcome an increased energy barrier, leading to an increase in the solid solubility in the α-Al matrix.

### 3.4. Mechanical Property

**Figure 8:**
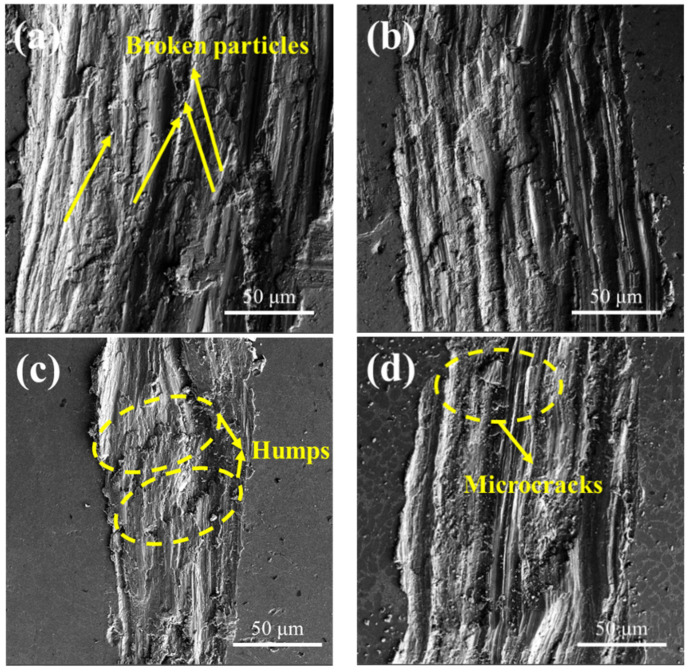
SEM morphology of wear surface under different frequency pulse conditions: (**a**) 0 Hz; (**b**) 500 Hz; (**c**) 1000 Hz; (**d**) 2000 Hz.

**Figure 9:**
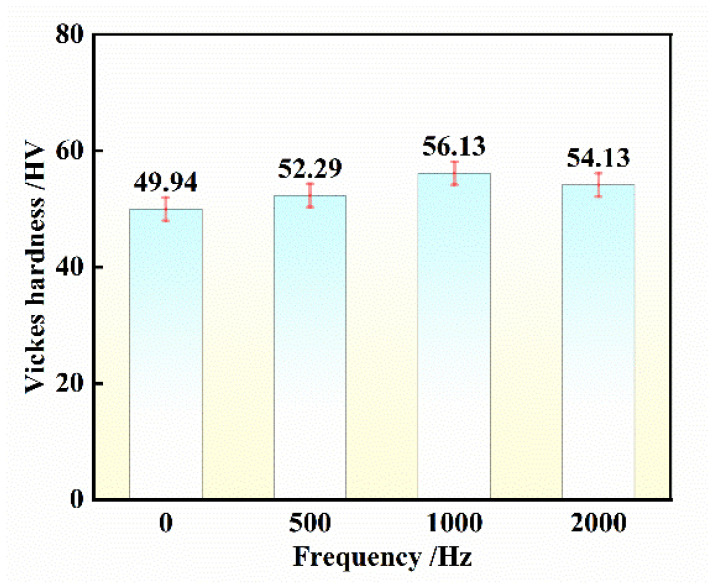
Changes in Vickers hardness of samples with different pulse frequencies.

The wear samples were cleaned with alcohol, and the wear surface morphology was observed under scanning electron microscopy, as shown in Figure 8. As seen in Figure 8a, many coarse broken particles and significant pits and cracks appeared on the material surface, indicating that the wear of the sample was primarily driven by chiseling furrows. In Figure 8b, after the 500 Hz pulse frequency treatment, the stratification phenomenon was alleviated, and the depth and width of furrows were reduced to varying degrees, along with a decrease in spalling into pits. Upon further increasing the pulse frequency to 1000 Hz, as shown in Figure 8c, the depth and width of wear marks were significantly improved, resulting in a visibly smoother wear surface with significantly reduced furrows and tunnels, although stratification still persisted. Furthermore, more carbon film was produced, improving wear-reduction performance. Combined with the microhardness results, as shown in Figure 9, five points were taken on the sample’s surface for hardness testing, and the average value was calculated with a 3 mm interval between each test point. The untreated material exhibited a low microhardness of only 49.94 HV. Consequently, micro-cracks occur when the shear stress exceeds the ultimate strength in the wear test. As wear friction progresses, micro-cracks gradually increase, ultimately leading to the formation of numerous massive cracks, representing a typical abrasive wear mechanism [29,30].

**Figure 10:**
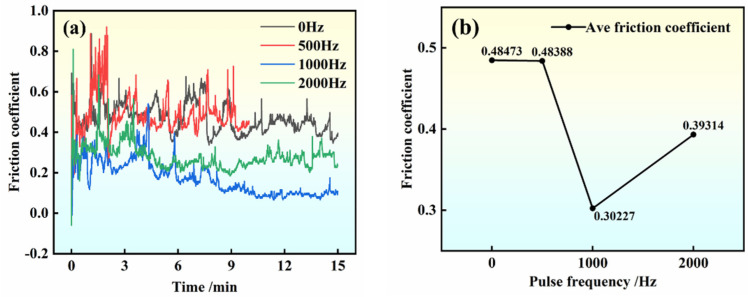
Friction and wear curves: (**a**) friction coefficient of samples with different frequencies; (**b**) average friction coefficient curve.

Figure 10 illustrates the change curve of the friction curve and friction coefficient of hypoeutectic Al-9Si alloy after treatment with different pulse frequencies. The friction coefficient reflects the contact state between friction and wear materials [31], and its magnitude is influenced by load, contact area, and lubrication [32]. It is observed that the friction curve of the sample following high-frequency pulse treatment exhibits a stable and gradual trend, with an average value of the optimal friction coefficient at 0.3 ± 0.01. In contrast, the average friction coefficient of the untreated sample is 0.6 ± 0.01. These results indicate that the wear resistance of the hypoeutectic Al-9Si alloy is improved after high-frequency pulse treatment compared to the original Al-9Si alloy. The Al-9Si alloy demonstrates enhanced wear resistance and can be utilized for surface protection in production and daily life. Furthermore, the friction coefficients of the 1000 Hz samples are lower than those of the 500 Hz and 2000 Hz samples, suggesting that the application of high-frequency pulses during the solidification process of hypoeutectic Al-9Si alloy can enhance wear reduction performance.

**Figure 11:**
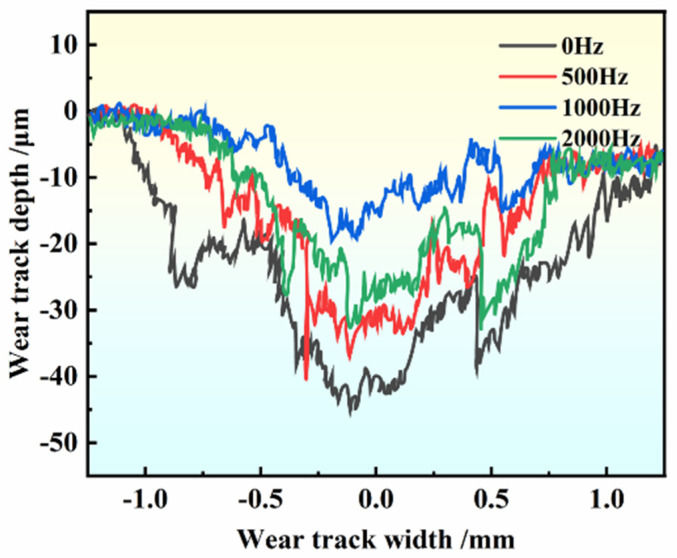
Wear track profiles of samples with different pulse frequencies.

Figure 11 shows the wear track of samples treated with pulse inoculation at different frequencies. It is evident from the figure that the average wear depth of untreated samples is 41 ± 3 μm, whereas that of samples treated with a high-frequency pulse is 25 ± 3 μm. The average wear track depth of the treated sample is lower than that of the base material. This can be attributed to the higher microhardness of the treated sample compared to the original sample. As demonstrated in Figure 7, increased microhardness of the material leads to enhanced resistance to hard ball pressing into its surface, resulting in reduced friction, smaller width of the annular wear surface, shallower depth, decreased contact area between the friction pairs, and shallower friction track [33,34]. Hence, aluminum alloys treated with high-frequency pulses exhibit superior wear resistance compared to untreated materials. The average wear volume of the treated material was 0.054 413 m^3^, while that of the original material was 0.168 05 m^3^. The wear rate after high-frequency pulse treatment was less than half that of the original material, indicating that the material after high-frequency pulse inoculation possesses higher wear resistance, consistent with the above conclusion.

## 4. Conclusions

This paper focuses on the effects of high-frequency pulses on improving the solidification structure and enhancing the mechanical properties of sub-eutectic Al-9Si alloys, and by observing the macroscopic structures, the following conclusions were drawn:

(1) In an argon gas atmosphere, high-frequency pulses were applied to induce grain refinement through a process known as incubation treatment. The dominant mechanism for grain refinement was attributed to the cluster theory and intense electromagnetic stirring. At a frequency of 1000 Hz, the grain size was measured at 13.87 μm. Further increasing the pulse frequency resulted in a larger re-melting area and grain size.
(2) After undergoing high-frequency pulse processing, the eutectic structure of Al-9Si transformed into fine and dense clusters, with nearly the complete elimination of primary Si. Spectral analysis revealed that increased pulse frequency led to a higher solubility of Si elements in α-Al.
(3) High-frequency pulses can enhance the comprehensive mechanical properties of sub-eutectic Al-9Si. The average friction coefficient is the smallest, the wear depth is the shallowest when the applied current density is 300 A/cm^2^, and the frequency is 1000 Hz when the material hardness is a maximum of 56.13 HV.

## Figures and Tables

**Table 1:** Hypoeutectic Al-Si alloy element content percentage (wt.%).

Element	Al	Zn	Cu	Mg	Others
Content	89.481	0.611	0.387	0.156	0.241

## Data Availability

Data are contained within the article.
